# Molecular mechanisms of the interplay between Vpx and SAMHD1

**DOI:** 10.1186/1742-4690-10-S1-P1

**Published:** 2013-09-19

**Authors:** Ying Wu, Maria DeLucia, Jennifer Mehrens, Jinwoo Ahn

**Affiliations:** 1 Structural Biology, University of Pittsburgh School of Medicine, Pittsburgh, PA, USA

## Background

SAMHD1, a dGTP-regulated deoxyribonucleoside triphosphate (dNTP) triphosphohydrolase, down-regulates dNTP pools in terminally differentiated and quiescent cells, thereby inhibiting HIV-1 infection at the reverse transcription step. HIV-2 and SIV counteract this restriction via a virion-associated virulence accessory factor, Vpx (Vpr in some SIVs), which loads SAMHD1 onto CRL4-DCAF1 E3 ubiquitin ligase for poly-ubiquitination, programming it for proteasome-dependent degradation. However, the detailed molecular mechanisms of SAMHD1 recruitment to the E3 ligase have not been defined. Further, whether divergent, orthologous Vpr/Vpx proteins, encoded by distinct HIV/SIV strains, bind SAMHD1 in a similar manner, at a molecular level, is not known.

## Materials and methods

We applied systematic biochemical and bioanalytical approaches to investigate molecular mechanisms of SAMHD1 recruitment to CRL4-DCAF1 E3 ubiquitin ligase in complex with Vpr/Vpx.

## Results

Vpx from the HIV-2 and SIVmac lineage, bound to DCAF1, interfaces with the N-terminus of SAMHD1, while Vpx from the SIVmnd2 and SIVrcm lineage requires the N-terminus of SAMHD1 for interaction, ubiquitination, and degradation. Interestingly, Vpr from SIVagm strains targeted both the N- and the C-terminus of SAMHD1. Real time kinetic analysis suggested that the viral proteins in complex with DCAF1, interacted with primate SAMHD1 proteins with nanomolar affinity, manifested by rapid association and slow dissociation, regardless of binding modes; the N-terminus or the C-terminus of SAMHD1. A single amino acid change at the binding interface of the host restriction factor is sufficient to change its susceptibility to degradation by the virulence factors from specific HIV/SIV strains. Further, we provide evidence that Vpr/Vpx binding to SAMHD1 inhibits its catalytic activity and induces disassembly of a dGTP-dependent SAMHD1 oligomer.

## Conclusions

The biochemical mechanism underlying viral-hijacking of the E3 ubiquitin ligase appears to be common among viral clades and to involve an increase in the time that cellular targets are resident at the substrate receptor for poly-ubiquitination. Furthermore, our studies reveal a previously unrecognized biochemical mechanism of Vpx-mediated SAMHD1 inhibition: direct down-modulation of its catalytic activity, mediated by the same binding event that leads to SAMHD1 recruitment to the E3 ubiquitin ligase for proteasome-dependent degradation.

